# Design Principles to Accommodate Older Adults

**DOI:** 10.5539/gjhs.v4n2p2

**Published:** 2012-03-01

**Authors:** Miranda A. Farage, Kenneth W. Miller, Funmi Ajayi, Deborah Hutchins

**Affiliations:** Feminine Care Innovation Center, The Procter & Gamble Company 6110 Center Hill Rd, Cincinnati, OH 45224, USA Tel: 513-634-5594 E-mail: farage.m@pg.com; The Procter and Gamble Company, Cincinnati, OH USA E-mail: miller.kw@pg.com; The Procter and Gamble Company, Cincinnati, OH USA E-mail: Ajayi.fo@pg.com; Hutchins & Associates LLC, Cincinnati, OH USA E-mail: hutchinsda@fuse.net

**Keywords:** Aging, Function, Sensory, Mobility, Cognition, Universal Design, Accommodation, Usability

## Abstract

The global population is aging. In many industrial countries, almost one in five people are over age 65. As people age, gradual changes ensue in vision, hearing, balance, coordination, and memory. Products, communication materials, and the physical environment must be thoughtfully designed to meet the needs of people of all ages. This article summarizes normal changes in sensory function, mobility, balance, memory, and attention that occur with age. It presents practical guidelines that allow design professionals to accommodate these changes and better meet the needs of older adults. Designing for older adults is inclusive design: it accommodates a range of physical and cognitive abilities and promotes simplicity, flexibility, and ease of use for people of any age.

## 1. Introduction

**Figure 1:**
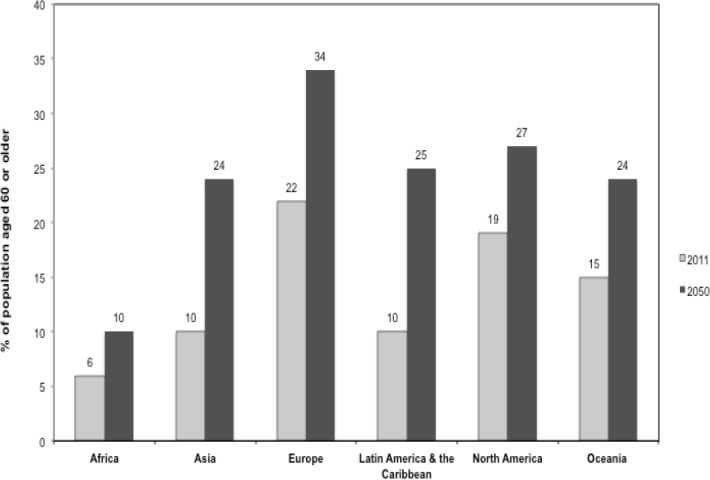
United Nations estimates of aging by region, 2011 and 2050 (projected) Statistics obtained from the United Nations, Department of Economic and Social Affairs, Population Division. World Population Prospects: The 2010 Revision, Highlights and Advance Tables (2011) (Working Paper No. ESA/P/WP.220). Retrieved February 20, 2012, from United Nations http://esa.un.org/unpd/wpp/

The global population is aging (Figure 1) (United Nations, Department of Economic and Social Affairs, Population Division. World Population Prospects: The 2010 Revision, Highlights and Advance Tables, 2011). In the developed world-where the trend is most pronounced-the generational focal point is about to shift from youth to old age. In a few decades, Japan will have the largest population of older people in the world: one of every five people in Japan is aged 65 or older and half of this group is over age 75. In Europe, close to one in five people is 65 or older (The US Census Bureau. World Population Information. World Population by Age and Sex, by Region, 2009). In the United States, for the first time in history, more people over age 64 than under age 25 and twice as many people are over age 64 than under age 5 (The US Census Bureau. USA statistics in brief. Population-Sex, Age, 2009). For perspective, the number of people aged 65 or older in the United States (almost 38 million) exceeds the entire population of Canada (33 million) (The US Census Bureau. USA statistics in brief. Population - Sex, Age, 2009; World Bank Data Finder. Total Population, 2010 (mid-year estimates, 2008).

This demographic shift will require more thoughtful design of products, packaging, media, information technology, workplace features, transportation, and public and private spaces to minimize hazards and better meet the needs of older adults. This article is intended to be a practical resource to assist designers and those in the helping professions to recognize and accommodate the needs of older people. Normal and expected changes in sensory function, mobility, and cognitive ability that accompany aging are described and practical guidelines accommodating these changes are presented. The age-related changes and recommended accommodation discussed herein are also summarized in a tabular reference format as a supplement to the text. We believe that professionals in many fields (product and technology development, education, communication, public policy, and the helping professions) can harness these guidelines to improve usability not only for older adults but also for people with a range of physical, sensory and cognitive abilities.

## 2. Search Methodology

Demographic information was obtained from online databases of the United Nations, the US Census, and the World Bank. The primary literature on functional changes associated with aging is voluminous. Consequently, the search strategy began by identifying published reference handbooks and technical reports on research in two areas: (1) functional changes associated with aging and (2) human factors design. These were obtained by searching online booksellers and the PubMed book database. Search terms included elders, older adults, elderly, seniors, aging, age, sensory, mobility, balance, memory, cognition, accommodation, universal design, transgenerational design, human factors, usability, and marketing. Special editions of journals in the fields of gerontology as well as magazines on usability specifically dedicated to these concerns were found with search engines such as Google and Google Scholar. Primary sources were obtained from the bibliographies of pertinent book chapters, technical reports, magazine articles, and journal articles; these were supplemented with online searches of PubMed and local University library catalogs using similar terms focusing on the last 10 years. Our goal for this article was to distill information from these sources into a set of data-based yet practical reference guidelines for accommodating the needs of older adults.

## 3. Age-related Functional Changes and Accommodation Guidelines

Who is the older person? The definition is as much cultural as it is chronological. For example, in the US, the American Association of Retired Persons (AARP) (aarp.org) solicits members aged 50 and up, irrespective of work status. Age 65 is a milestone enshrined as the age of retirement in the current legal framework of the US Social Security system. In medical research, people aged 18–44 are designated as adults, those 45–64 are considered middle-aged, and those over 65 are aged (www.ncbi.nlm.nih.gov). Consequently, this article focuses not on chronological age, but on the physical and functional changes that ensue over time – changes that prompt individuals’ reassessment of their capabilities and of the ways in which they interact with others and with the physical environment.

The following subsections describe normal and expected changes in sensory function, mobility, and cognition that develop as people age and practical guidelines for accommodating these changes. The focus is on functional changes that affect the usability of articles, the ability to negotiate the physical environment, the effective presentation of communication materials, and safety considerations for an aging population. It is important to recognize that good design for older adults is often good design for everyone: these accommodation guidelines are consistent with the principles of Universal Design (Table 1), which promote simplicity and equity for people with a range of abilities, irrespective of age (Gassman & Reepmeyer, 2008). As practical aid to interested readers, the research discussed in this review is summarized for easy reference in supplemental tables at the end of the text (Tables S1 through S9).

**Table 1:** Universal design principles

PRINCIPLE	EXPLANATION
**Equity**	Useful and appealing to people with diverse abilities Used in the same or equivalent way by everyone Does not segregate or stigmatize anyone

**Flexibility**	Accommodates a range of preferences and abilities Provides choice in method of use Facilitates accuracy and precision Adapts to user constraints and pace

**Simplicity**	Easy or intuitive regardless of experience, knowledge base, language skills, and level of concentration ○ Consistent with users’ expectations ○ Eliminates complexity ○ Provides cues, prompts, and feedback

**Perceptibility**	Communicates necessary information regardless of ambient conditions and the expected range of user sensory ability ○ Redundant modes of presentation through different senses (verbal, written, auditory, tactile) ○ Highlights essential information ○ Differentiates elements ○ Maximizes legibility ○ Compatible with glasses, hearing aids, walkers, etc

**Error recovery**	Minimizes hazards and unintended actions ○ Useful elements are accessible, hazardous elements are sequestered or eliminated. Fail-safe features incorporated ○ Warning signals ○ Not dependent on vigilance

**Low effort**	Can be used comfortably with minimal strain or fatigue ○ Minimizes force or sustained effort ○ Minimizes repetitive actions ○ Requires neutral body positions

**Accessibility**	Accessible for expected range of body size, posture, or mobility Clear sight lines Comfortable reach Accommodates variations in hand grip or size Minimizes physical barriers

Adapted from: Gassman, O., & Reepmeyer, G. (2008). Universal Design - Innovations for all ages. In: F. Kohlbacher & C. Herstatt (Eds.), The Silver Market Phenomenon. Chapter 9 (pp. 125-148). Berlin and Heidelberg: Springer Verlag.

**Table:** Table S1. Age-related changes in vision

CHANGES IN VISUAL FUNCTION	DESCRIPTION AND CAUSE	IMPACT
**Impaired near focus** (presbyopia)	Cannot focus on nearby objects. Caused by lens stiffening. Begins at age 40.	Need reading glasses or bifocal correction. Near point focus is 10 cm at age 20, 100 cm at age 70.

**Reduced acuity** (sharpness)	Sharpness of focus declines. Less light enters the eye. Begins at age 50.	More illumination is needed to see sharply. A 60-year-old with normal vision needs twice the level of illumination as a 20-year-old.

**Sensitivity to glare**	Light scatters in the eye due to changes in the vitreous humor and cataracts. Halos might be seen around a light source.	Vision is impaired by direct light, reflected light, glossy paper, reflective wall coverings, highly polished floors, lack of window shades.
**Brightness adaptation**	Slower adaptation to changes in light levels, especially with cataracts.	Difficult to adjust to indoor and outdoor light or to rapidly changing visual stimuli.
	Harder to distinguish dark and light surfaces.	Higher contrasts needed for reading.

**Darkness adaptation**	Light perception threshold increases, doubling each 13 years between age 20 and age 60 (fewer rods in the eye).	Need greater levels of illumination (without glare) and higher contrasts for clarity.
	Light gathering power at 60 is one-third that at 20.	Hard to perceive motion at low illumination. Night driving becomes difficult.

**Color perception**	Can’t distinguish violet, blues and greens (lens and vitreous yellowing).	Older people prefer warm colors to cool colors.
	Color brightness and purity perception diminishes.	Elder color preferences can be exuberant; may seem garish to the young.

**Visual field narrowing**	At age 35, circular visual field is 180º horizontal 135º vertical. Declines begin at 40.	Eyeglasses do not add peripheral vision.
	Reduced peripheral vision (*tunnel vision)*.	Compensatory head turning, greater vigilance, or assistance may be required.

**Table:** Table S2. Visual presentation guidelines

VARIABLE	ACCOMMODATION PREFERENCE	DESIGN AND PRESENTATION CHOICES
**LIGHTING**	**BETTER**	Raise Illumination On Reading Surfaces To 100 Cd/M^2^ Reflected Light. Use Several Small, Low-Intensity Light Sources Instead Of A Single Large One. Diffuse Light.
**GLARE**	**BETTER**	Use Matte Surfaces.
	**WORSE**	Text Or Print On Glossy Paper.

**COMPOSITION**	**BETTER**	Stimuli Needing Inspection Should Be Large, Simple, Uncrowded, And In The Central Visual Field. Make Things Conspicuous Through Size, Color, Or Contrast. Place Task-Specific Information On A Constant Depth-Of-Plane.

	**WORSE**	Busy Backgrounds Or The Need To Discriminate At Close Distances.

**TEXT**		

**FONT TYPE**	**BETTER**	Sans Serif: • Arial • Helvetica • Century Gothic Serif: • Times • Bookman • Book Antigua
	**WORSE**	○ Script Fonts Are Difficult To Read ○ Decorative Fonts Are Difficult To Read

**FONT SIZE**	**BETTER**	Minimum 12 Pt Font: Arial • Helvetica • Century Gothic Times • Bookman • Book Antigua

**FONT CASE**	**BETTER**	**UPPERCASE DRAWS ATTENTION**
	**WORSE**	BUT SHOULD NOT BE USED FOR LONG BLOCKS OF TEXT

**CONTRAST**		

	**BETTER**	Black Text On White Background
		**WHITE TEXT On Black Background**
		○Try To Achieve 50:1 Contrast (E.G. LCD Screen)
**COLOR**	**BETTER**	Warm Colors. (Use Larger Contrast Steps If Short Wavelength Discrimination Needed.)
	**WORSE**	Signaling Information With Violet-Blue-Green (Difficult To Perceive)

**MOTION / 3-D**	**WORSE**	Rapidly Changing, Flickering Or Moving Stimuli; Virtual Reality

**Table:** Table S3. Age-related changes in hearing

CHANGES IN AUDITORY FUNCTION	DESCRIPTION AND OR CAUSE	IMPACT
**Sound perception and speech recognition**	As background, • Normal threshold is 0-3 dB. • Conversational speech is 50 dB. • Pain threshold is 100 dB. Hearing loss is 2.5 dB per decade to age 55, 8.5 dB per decade beyond.	Low-level sounds are muffled. 25% percent loss in ability to perceive normally audible speech by age 60. Deficit is small in quiet environments but difficulty increases when discriminating from background noise. Older adults use context cues to aid speech recognition.

**Frequency (pitch) discrimination**	Normal speech ranges from 500 Hz to 2000 Hz. Perception of high frequencies diminishes with age, especially in men.	Hissing or ringing in the ear (tinnitus). Words with high-pitched consonants (c, ch, f, s, sh and z) more difficult to comprehend.

**Identifying sound location**	Both ears needed to locate sound.	Stimulus to one ear vs. another can cause disorientation.

**Auditory attention**	Older adults have difficulty disregarding competing information reaching one ear versus another. Hard to process distinct sounds at rapid rates.	Affects performance in multi-task environments. Recall drops as speech rates increase.

**Table:** Table S4. Auditory presentation guidelines

VARIABLE	ACCOMMODATION PREFERENCE	PRESENTATION CHOICES
**SOUND INTENSITY**	**BETTER**	• Sound signals should be at least 60 dB at the ear of the listener. (Conversational speech is 50 dB.) • Allow for volume adjustments. Use simple instructions and controls with simple movement (e.g. back and forth along graduated level rather than turning knob)

**SOUND FREQUENCY**	**BETTER**	• Use sound alerts within frequency range of 500 to 2000 Hz. • Male voices are better than female for announcements.
	**WORSE**	• High frequencies (avoid frequencies above 4000 Hz) • Artificial (synthesized) speech

**DISCRIMINATION OF SOUND CUES**	**BETTER**	• Maximize pitch, spectral or location differences of individual sound cues.

**SOUND LOCATION**	**BETTER**	• If you must signal sound location at high frequency (>2000 Hz), use longer duration (>0.5 s)

**AUDITORY ATTENTION**	**BETTER**	• Provide redundant cueing through cross-sensory channels (e.g. augment a sound signal [cell phone ring] with vibration; sound alarm with light.) • Provide headphone sets for focused work during group training.

	**WORSE**	• Background noise or reverberation. (Need to maintain high “signal-to-noise” ratio)

**SPEECH RECOGNITION**	**BETTER**	• Reasonable pace • Predictable linguistic structure • Pauses at grammatical boundaries (after phrases, end of sentences)
	**WORSE**	• Synthesized or robotic speech patterns • Over-exaggerated and patronizing simplicity (‘*elderspeak*’; ‘*mother-ese*’)

**Table:** Table S5. Age-related changes in touch and temperature perception, mobility, and balance

AGE-RELATED CHANGES	DESCRIPTION/CAUSE	IMPACT
**Pressure sensitivity**	Perception of contact may diminish.	Harder to sense depression of keyboard keys, placement of body against a surface.

**Thermal sensitivity**	Reduced nerve ending function and less heat retention.	Heightened sensitivity to low temperatures. May not react quickly to dangerously high temperatures.
**Body changes**	Range of motion decreases. Trunk height decreases. Sitting height decreases. Arm reach decreases.	Ergonomics for average adult does not suit older people.

**Slower movement and reflexes**	Loss of muscle strength and tone. Declines in neural responses.	Deliberate movement is slower, as is reaction to stimuli. Pursuit of targets (e.g. tracking with a computer mouse) is slower.

**Flexibility**	Muscles are stiffer and less limber	More limited stretch and reach.

**Arthritis and Tremor**	Arthritis causes joint pain and stiffness. It affects 50% of seniors and 80% of elders. Some tremor occurs with aging. Parkinsonism, a neurological ailment, causes tremor and affects 1% of those over 50.	Difficult to grip with the hands or to bend the joints, which interferes with performance of everyday tasks (e.g. holding a rail, unscrewing a bottle cap.) Precise, targeted motion is difficult (dialing a phone, inserting an ATM card, setting controls on appliances.)

**Coordination**	Ability to time and execute movement in a coordinated way is reduced.	Difficult to produce precisely timed sequences of movements (e.g. rapid double click of a computer mouse.) Older people move with deliberation (they trade speed for accuracy).

**Balance**	Diminished static postural control. Diminished dynamic balance.	Walking speed slows 10% – 20% per decade beyond age 60. Risk of falls increases with age. Older people walk cautiously (they trade speed for balance).

**Table:** Table S6. Accommodation guidelines for diminished temperature and touch sensation

AGE-RELATED CHANGES	ACCOMMODATION PREFERENCE	DESIGN AND PRESENTATION CHOICES
**TEMPERATURE SENSATION**	**BETTER**	• Maximum residential hot water temperature is 120ºF (48.9ºC). • Use of heating pads should be weighed against potential for burns. • Use supplemental sensory cues to warn of high temperatures (e.g. lighted red “H” on or next to an electric burner as long as it remains hot) • Where high temperatures cannot be signaled, incorporate protective measures (e.g. cookware with stay-cool handles) • Keep homes at a minimum temperature of 65ºF (18ºC).
**TOUCH AND PRESSURE SENSITIVITY**	**BETTER**	• Add supplemental sensory cues: ○ Textured rather than smooth surfaces supplement touch sensation. ○ Audible sounds reinforce that a button or computer key had been depressed.

**Table:** Table S7. Accommodation guidelines for movement restriction and balance instability

AGE-RELATED CHANGES	ACCOMMODATION PREFERENCE	DESIGN AND PRESENTATION CHOICES
**BODY CHANGES (STATURE, REACH, ETC.)**	**BETTER**	• Shelve products lower. • Chairs should seat near center of gravity and allow feet to be placed on the floor. (Many chairs are too low.)

**FLEXIBILITY**	**BETTER**	• Account for limitations in stretch and reach in product shelving.

**SLOWER MOVEMENT AND REFLEXES**	**BETTER**	• Allow time for discrete tasks.
	**WORSE**	• Multiple rapid steps (e.g. adjust double-click speed of computer mouse, tracking speed on scroll ball)

**COORDINATION AND TREMOR**	**BETTER**	• Simple task movements • Levers are better than knobs that must be turned. • Large caps are better than small. • Well-designed flip-tops may be better than screw tops. • Large buttons rather than small ones. • Things should “snap” into position.
	**WORSE**	• Failing to guard against accidental actuation of critical controls.

**BALANCE**	**BETTER**	• Provide rails, grip bars. • Use solid color carpets. • Use contrasting strips and paint to distinguish surfaces (e.g. edges of stairs, walls from door frames or floors). • Avoid shiny surfaces and glare.
	**WORSE**	• Unsecured objects (e.g. unsecured end-of-aisle displays present obstacles to motorized or static ambulatory aids.) • Patterned carpets, shiny floors. • No contrast between horizontal and vertical surfaces or surfaces in different planes. • Absence of handrails.

**Table:** Table S8. Age-related changes in memory and cognition

FUNCTION	DESCRIPTION	IMPACT OF AGE
**Working memory**	Active memory of what is presently perceived and thought about. Negligible age differences for simple procedures.	Fewer discrete information bits can be processed in a given time. Recall decays faster. Information overload is overwhelming.

**Prospective memory**	Remembering to do something in the future (e.g. at a particular time; after an elapsed time; or in response to an event.)	Much greater age declines for recalling future time-based tasks (take pill after 4 hours) than for event-based tasks (when buzzer sounds, turn off the oven.)

**Semantic memory**	Long-term repository of world knowledge (vocabulary, rules of language, concepts, history, culture, art, music, etc.)	No deficit with age. The recognized expert is often an older person. Temporary blocks in retrieval (“tip of the tongue” experiences) increase with age, but information repository and organization is intact.

**Procedural memory**	Knowledge about how to do things. Some knowledge is virtually automatic (steering a car, shifting gears.) Some relates to explicit but well-practiced routines (following a recipe; using a word processor.)	Automatic behaviors remain largely intact. Previously well–learned procedures are harder to inhibit in a new context (e.g. when faced with inconsistent on/off positions.) Older people can learn new skills (e.g. using a spreadsheet) but require more time to do so.

**Attention**	The capacity to focus on and process information.	Takes longer to orient attention from one thing to another. Less able to inhibit irrelevant information. Speed and multi-tasking (e.g. looking for street signs while driving) are a challenge.

**Spacial cognition**	Using external visual cues to mentally orient in three-dimensions (using a map to traverse a physical space)	Mentally transforming spacial information, developing sequences, becomes more difficult with age.

**Language comprehension**	Interpreting verbal and written information and drawing inferences	Subtlety, irony, and unfamiliar context inhibit drawing inferences.

**Table:** Table S9. Accommodating age-related cognitive changes

FUNCTION	ACCOMMODATION PREFERENCE	DESIGN AND PRESENTATION CHOICES
**WORKING MEMORY**	**BETTER**	• Simple instructions – discrete short messages • Label icons; pictorial info should be highly intuitive.
	**WORSE**	• Information overload
**PROSPECTIVE MEMORY**	**BETTER**	• Provide event-based reminders (e.g. voice mail reminders; buzzer sounds – but with caution, see below).
	**WORSE**	• Time-based instructions (e.g. take pill every four to six hours) • Too many similarly-beeping gadgets

**PROCEDURAL MEMORY**	**BETTER**	• Simple intuitive steps. Place in context. • Minimize the number of steps. • Slow the pace of training. Frequent repetition for reinforcement. Practice opportunities. • Give feedback cues for correct action (a key “clicks” or “beeps” when depressed.)
	**WORSE**	• Complex, multi-step process. • Procedures inconsistent with established practice.

**ATTENTION**	**BETTER**	• Simple displays. Short, discrete signals.
	**WORSE**	• Avoid visual clutter or background noise. • Avoid arrays of functions and displays. • Avoid concurrent actions (pressing three keyboard keys to execute command.)

**MESSAGE COMPREHENSION**	**BETTER**	• Clear messages • Reasonable pace • Predictable linguistic structure • Pauses at grammatical boundaries (after phrases, end of sentences) • Redundant information • Rich in context
	**WORSE**	• Having to process several pieces of information to draw a conclusion (e.g. typical automated telephone prompts) • Presenting irrelevant information • Subtlety, irony, ambiguity • Unfamiliar context

### 3.1 Changes in Visual Function

One of the earliest signs of aging is the impairment of near-focus, or presbyopia. This results from a stiffening of the lens of the eye such that it no longer accommodates sufficiently to allow focus on near objects. Most people first notice this impairment in their early to mid-40s. The near point focus is 10 cm at age 20, compared to 100 cm at age 70 (Sloane, 1992; Sun *et al.*, 1988). Presbyopia is corrected with reading glasses or bifocal lenses.

As the pupil becomes smaller and less light is able to enter the eye, more illumination is needed in order to see sharply. This need increases progressively with age, such that a 60-year old with normal vision needs twice the illumination to see sharply as a 20-year old (Pirkl, 1994; Weale, 1961). However, illumination must be controlled in such a way as to reduce glare (Vos, 1995). Older adults are more sensitive to glare because changes in the condition of the vitreous humor (and, in some cases, cataracts) create light scattering within the eye.

Older people also adapt more slowly to changes in illumination (D. B. Elliott & Whitaker, 1991). It takes time to adjust from indoor to outdoor light, and vice-versa. Bright lights, such as oncoming headlights, are bothersome.

Higher contrasts are needed to distinguish surfaces or to discriminate between objects (e.g. between the edge of one step and the step below it) (D. Elliott, Whitaker, & MacVeigh, 1990; Scialfa, Adams, & Giovanetto, 1991; Scialfa, Garvey, Tyrrell, & Leibowitz, 1992). It becomes difficult to adjust to rapidly changing visual stimuli (D. Kline, Culham, Bartel, & Lynk, 1994; Warren, Blackwell, & Morris, 1989) and to perceive motion at low illumination. Night driving becomes more difficult (D. W. Kline *et al.*, 1992).

Color perception diminishes, especially in the violet-blue-green potion of the spectrum (Johnson, Adams, Twelker, & Quigg, 1988), such that vivid shades of color in the yellow to red portion of the spectrum are more easily discerned. Consequently, older people discern and discriminate bright, warm colors more easily than cool ones (Wijk, Berg, Sivik, & Steen, 1999).

Lastly, the visual field narrows so that peripheral vision declines (Collins, Brown, & Bowman, 1989). The field of vision within which it is possible to identify and discriminate visual targets relevant to a task (without compensatory eye and head movements) is known as the useful field of view (UFOV). Considerable evidence exists that the useful field of view is restricted in older adults (Ball, Beard, Roenker, Miller, & Griggs, 1988; Edwards *et al.*, 2006; Sekuler, Bennett, & Mamelak, 2000) and older adults must compensate for this by heightened vigilance and by scanning the environment with more deliberation.

For extensive reviews of age-related changes in vision, the reader is referred to vision (D. W. Kline & Scialfa, 1997; Pirkl, 1994; Stuen & Faye, 2003). A summary of normal age-related changes in vision can be found in Supplemental Table S1.

### 3.2 Visual Presentation Guidelines

Simple guidelines for accommodating age-related visual impairments will increase perceptibility for people of any age with a range of sensory activity.

Higher illumination without glare can be achieved with more numerous low intensity sources or with diffuse rather than direct sources of light (recessed lighting, sheer curtains, lampshades). The illumination of reading surfaces should be 100 cd/m^2^ reflected light. To minimize glare, matte rather than glossy surfaces should be used for reading materials and in the physical environment.

High contrast (50:1) on-screen and in print (such as white text on a black background, or black text on a white black background) facilitates legibility. Color choices in the long-wavelength end of the spectrum (“warm” colors) are preferred: if short-wavelengths (“cool” colors) are used, large contrast steps will be required to facilitate perceptibility. Shades of any color used to convey information must be clearly distinct from the background.

Simplicity of visual presentation is key: visual clutter should be avoided. Important information should be large, conspicuous, uncrowded, and in the central visual field. In print materials, font sizes of 12-points or larger are preferred; decorative fonts or backgrounds should be avoided. Uppercase is useful to highlight key material, but is tiring to the reader when used in long blocks of text.

A substantial number of older adults use computers and the Internet to connect with family and friends, to do online shopping, to join social networks, and to link to the world of information (Fisk, Rogers, Charness, Czaja, & Sharit, 2009f; Willis, 2006). Software that allows the user to adjust and enlarge text and graphics, as well as voice output software with clear enunciation, make computer use easier for the visually impaired. Distracting visual stimuli (such as elaborate backgrounds, flashing lights, rapid motion, or flickering) should be avoided unless used judiciously to signal a specific, needed action. For a more in-depth discussion on interface and multimedia design, the reader is referred to (Fisk, Rogers, Charness, Czaja, & Sharit, 2009d; Fisk *et al.*, 2009f; Fisk, Rogers, Charness, Czaja, & Sharit, 2009h).

For comprehensive reviews of visual presentation guidelines, the reader is referred to extensive reviews of human factors design (Fisk, Rogers, Charness, Czaja, & Sharit, 2009e; D. W. Kline & Scialfa, 1997). The accommodations discussed in this section are summarized for reference in Supplemental Table S2.

### 3.3 Changes in Hearing

The ability to perceive pure tones and low intensity sound diminishes with age, a sensorineural impairment known as presbycusis. In a young person, the normal sound threshold is 0-3 decibels (dB). Sound perception diminishes by 2.5 dB per decade up to age 55, and then accelerates to 8.5 dB per decade thereafter (Davis, Ostri, & Parving, 1991). Sixty percent of people aged 55 or older have some form of hearing impairment and 20% of those over age 80 require a hearing aid (Davis, 1991).

Perception of high-pitched sounds diminishes, especially in men (Pedersen, Rosenhall, & Moller, 1989) and exposure to high-pitched sound causes hissing or ringing in the ear (tinnitus). Words with high-pitched consonants (e.g. ch, sh, z) are harder to discern.

By age 60, there is a 25% loss in ability to perceive conversational speech. Speech recognition may be affected by a diminished ability to process the gaps between dynamic sounds that aid understanding (Lister, Besing, & Koehnke, 2002; Lister & Tarver, 2004; Pichora-Fuller, Schneider, Benson, Hamstra, & Storzer, 2006). Older people also experience difficulty discerning distinct sounds delivered in succession (Fitzgibbons & Gordon-Salant, 2010; Fitzgibbons, Gordon-Salant, & Barrett, 2007). Older adults rely on sound reaching both ears for clear perception and find it difficult to disregard competing auditory information (Espinoza-Varas & Jang, 2006). As a result of these changes, both speech recognition and discriminating speech from background noise become difficult (van Thriel *et al.*, 2006). A common complaint is, “I can hear people talking, but I can’t make out the words”. Hence, older adults often rely on lip reading and context cues to understand what is being said.

People who are over age 70 are more likely to have some degree of both vision and hearing loss. One study indicated a 13% prevalence of dual impairment in those aged 70-74, 27% in those aged 80-84, and 40% in those aged 85 or older (Brennan, Horowitz, & Su, 2005). Dual impairment hinders independence: it affects personal activities (such as walking, sitting and rising, and getting around in unfamiliar places) as well as activities requiring the use of instruments or appliances (such as preparing meals, going shopping, and using a telephone). Adults with dual impairments rely on informal help, formal assistance, or may live with others to obtain support.

Detailed reviews of age-related changes in hearing are presented in (D. W. Kline & Scialfa, 1997; Pirkl, 1994; Weinstein, 2003). Age-related auditory changes are summarized in Supplemental Table S3.

### 3.4 Auditory Presentation Guidelines

To aid auditory perception and recognition by older adults, sound signals of at least 60 dB should reach the ear (conversational speech is 50 dB). A high “signal-to-noise” ratio is necessary: the intended sound or message should be at a high enough volume with background noise kept to a minimum. Volumes should be adjustable.

Sound frequencies in the range of 500–2000 Hz are preferable. High frequencies should be avoided in both verbal and non-verbal auditory information: for example, lower-pitched male voices may be preferable to high-pitched female voices for announcements. Alarm sounds or other auditory cues should not exceed 2000 Hz.

An auditory signal can be reinforced by redundant cueing through another sensory channel (e.g. a telephone ring accompanied by vibration; a buzzer alarm accompanied by a flashing light). If different sound cues must be used to convey information, these should be from different parts of the sound spectrum and be distinctly spaced in time. Altering the location from which sound is emitted can also help to distinguish various sound cues. However, auditory signals and cues should be used judiciously. Multiple appliances that emit similar, high-pitched alarms plague modern households and confuse older users.

Verbal information should have a predictable linguistic pattern with expected pauses at grammatical boundaries (e.g. following parenthetic phrases or at the end of sentences). A slower pace of delivery aids the recognition and recall of verbal information, but the tone and pace of the delivery should be respectful and non-patronizing. Robotic, synthesized speech hinders comprehension.

In conversation with an older adult, speak clearly but in a non-exaggerated fashion. Use short sentences; pause slightly after each statement to facilitate comprehension by listeners who rely body language or other context cues to overcome hearing difficulties. Explicitly announce a new topic. If someone asks you to repeat what has been said, repeat once and then rephrase in slightly different way. Ask questions to be sure you have been understood. Do not assume that a head nod signals complete understanding.

For detailed discussion of the research on effective auditory presentation, the reader is referred to (Fisk *et al.*, 2009e; D. W. Kline & Scialfa, 1997). Guidelines for presenting auditory information are summarized for reference in Supplemental Table S4.

### 3.5 Changes in Odor and Flavor Perception

The Epidemiology of Hearing Loss Study, a US population-based study of sensory loss and aging in 2491 adults aged 53 to 97, found the prevalence of olfactory impairment (defined as correctly identifying fewer than 6 of 8 natural odorants commonly found in the home, such as chocolate or coffee) to be 24.5%, rising to 62.5% among those aged 80 or older (Murphy *et al.*, 2002). Men consistently demonstrated a higher prevalence of olfactory impairment in each age group. The National Social Life, Health and Aging Project (NSHAP), a US population-based study of community-dwelling older adults aged 57 to 85, found the prevalence of severe olfactory dysfunction (defined as correctly identifying ≤ 1 of 5 odorants: rose, leather, orange, fish, peppermint) to be 2.7% (women, 2.2%, men, 3.2%) (Boesveldt, Lindau, McClintock, Hummel, & Lundstrom, 2011). Odor identification declined with age in both sexes; peppermint odor was correctly identified most often (91.6%) and leather least often (70.5%).

The impact of age on the perception of chemical hazard odors is less well understood. People with olfactory dysfunction might be less likely to detect warning odors, such as the smell added to natural gas to alert homeowners of potential leaks. However, little systematic research on aging and perception of chemical odors was found. In the occupational setting, it is known that chemosensory thresholds vary dramatically among different types of industrial chemicals. An occupational health study of the chemosensory thresholds of various chemical classes (carboxylic acids; amines; esters; ketones and alcohol; and inorganics such as ammonia and hydrochloric acid) found large logarithmic differences in their odor perception thresholds and irritation thresholds. For example, the smell of an acrid substance like ethyl acrylate is first perceived at 6 × 10^-6^ ppm, compared to acetic acid (vinegar) at 0.59 ppm, and ethyl formate at 30 ppm (van Thriel *et al.*, 2006). These investigators did not find significant age-related differences in odor perception thresholds; small differences were determined only for acetic acid, propionic acid and acrylic acid. Mean odor thresholds in younger adults (18-35 years) were 0.37, 0.21 and 0.21 ppm for acetic, propionic and acrylic acids, respectively; in older adults (≥45) they were 0.67, 0.42 and 0.55, respectively.

In the domestic setting, common sense safety precautions are warranted. Because older people may be less likely to detect spoilage by smell, foodstuffs should be clearly labeled “Use before” or “Discard after” a specific date (Rawson, 2003). Bottles of household cleaners and chemicals should be labeled with large, bright, high contrast letters, and with conspicuous and recognizable warning symbols. Additional research is needed to assess the impact of age on olfactory perception of potential chemical hazards and to recommend appropriate guidelines to mitigate such hazards in the domestic or institutional settings.

The sense of taste is also depressed in older adults. The NSHAP found the prevalence of severe gustatory impairment (defined as correct identification of ≤1 of the four flavors: sour, bitter, sweet, salty, presented in that order) to be 14.8% (women 10.2%, men 19.2%) (Boesveldt *et al.*, 2011). The ability to perceive sweetness was preserved to the greatest degree, being correctly identified by 86.7% of subjects, whereas sour taste was correctly identified by only 39.4% of subjects. By contrast, an earlier review of published studies concluded that salt and bitter taste acuity declines with age, but that sweet and sour perceptivity does not (Winkler, Garg, Mekayarajjananonth, Bakaeen, & Khan, 1999).

Age-related declines in taste and smell may reduce the pleasure of cooking and eating. Medication, chronic disorders, and radiation therapy also alter taste perception (Hess, 1997). When food becomes tasteless and unappetizing, poor nutrition can result. Older adults seem to have a higher preference for salt than younger ones, and require double the salt content to perceive saltiness (Stevens, Cain, Demarque, & Ruthruff, 1991). Studies suggest a lower preference for foods with a predominantly sour taste (e.g. citrus fruit) or pungency (e.g. horseradish) and a higher preference for—and higher intake of—sweets and fats (Winkler *et al.*, 1999). This is perhaps not surprising, as enjoyment of sweet flavors is almost universal and the perception of sweetness is least affected by age. The chemosensory mechanisms for detecting palpable sensations, such as the burn of hot peppers, the tingle of carbonation, or the cooling of menthol, are the least affected by age (Rawson, 2003).

Notably, the ability to smell an aroma also affects the perception of flavor. Because odor perception diminishes with age, food may no longer taste as expected, especially if the perception of some aromatic components is affected more than others. Adults with a poor sense of smell are less likely to vary food selection, compromising nutritional content.

Evidence also exists that the food preferences of older adults are affected by texture: when soups of different thicknesses were presented to panels of older (>65 years) and younger adults (20-35 years), the older panel’s preference decreased as soup thickness increased, but the younger panels preference was unaffected (Forde, Cantau, Delahunty, & Elsner, 2002). Moreover, flavor and food preferences are regionally and culturally determined and will depend on the individual’s background and life experience. For example, older Americans who are independent and financially secure, and who have cultivated good nutrition habits during their lifetime, consume more varied and healthful diets than many young adults (Rolls, 1999). Because odor and flavor perception and preferences are so variable, no consistent guidelines on enhancing food flavor and appeal to older people have emerged. It may be that individualized strategies to improve appeal and palatability must be developed. For example, testing the appeal of more intensely flavored herbs and seasonings (e.g. rosemary, mint, dill, ginger, garlic, clove, chili, curry, etc.), and varying the texture and color of foods, may help add flavor complexity and variety to meals.

### 3.6 Changes in Touch and Temperature Perception, Mobility, and Balance

The techniques of quantitative sensory testing, which measure the threshold at which a subject perceives a defined, measurable stimulus, have been used to asses the impact of age on the perception of heat, cold and mechanical stimuli (i.e., touch, pressure and vibration) (Bartlett, Stewart, Tamblyn, & Abrahamowicz, 1998; Hilz *et al.*, 1998; Kenshalo, 1986; Lin, Hsieh, Chao, Chang, & Hsieh, 2005; Merchut & Toleikis, 1990). Research on thermal perception, though somewhat limited, suggests that older people are more sensitive to the cold and react more slowly to extremes in temperature.

The perception of touch, pressure, and vibration declines with age, especially on the hands and feet. Lower sensitivity to pressure makes it is harder to sense when the body has made full contact with a surface (such being fully seated) or when a small surface (such as an elevator button or keyboard key) has been depressed. The decline in sensitivity to touch, pressure, and vibration becomes apparent by the fifth decade and progresses exponentially after age 65 or 70 (Bartlett *et al.*, 1998; Hilz *et al.*, 1998). The rate of decline differs by body site. The perception of touch and vibration on the face remains relatively unchanged. By comparison, the sensitivity to touch on the hands and feet declines rapidly (Dyck, Karnes, O’Brien, & Zimmerman, 1993); this decline is more severe for the lower extremities (Kenshalo, 1986; Lin *et al.*, 2005), which may reflect the longer neural pathway that the sensory input must travel.

The impact of age on mobility is evident to the casual observer (Fisk, Rogers, Charness, Czaja, & Sharit, 2009a). The range of body motion is restricted, muscle strength declines, and the body is less flexible. Reflexes are slower. Trunk height and arm reach decrease, making it harder to reach for things (Kroemer, 1997). Some conditions, such as arthritis, cause joint pain and stiffness, making it difficult to grip and hold various surfaces.

Balance instability is an important issue (Newton, 2003). Static balance (when sitting still or when standing) and dynamic coordination (when negotiating the physical environment) are both compromised with age. Balance depends on three factors: (1) vision (to provide information about the location, dimension, and distinctness of objects); (2) the vestibular system of the inner ear (to provide information about the movement of the body through space); and (3) proprioception (feedback from receptors in the body about surfaces with which we are in contact) (Tideiksaar, 2002). Muscle weakness due to inactivity also contributes to a loss of balance.

Older people try to rely on vision to compensate for losses in the vestibular activity or proprioception, but the environment may work against them. For example, the glare of sunlight can make shiny floors a prime location for falls. Older people also walk and change positions more slowly, as they reduce their speed to help maintain balance and control (Vercruyssen, 1997).

The ergonomics of the physical environment challenge older people: for example, sitting positions may be too high for an older person’s center of gravity; product placements may be beyond the arm reach of the older person; turning a knob or opening a cap becomes difficult; pressing keys or buttons with adequate pressure and precision requires deliberation; rapid or combined sequences of movement (e.g. when needed to open a package, to actuate an appliances, or to perform a task on a computer) may be challenging. Older people may need to grasp handles, handrails or counters to maintain balance as they perform tasks.

Age related changes in touch and temperature perception, mobility, and balance discussed in this section are summarized in Supplemental Table S5.

### 3.7 Accommodating Altered Touch and Temperature Perception and Restricted Mobility and Balance

Accommodating changes in temperature and touch perception and restrictions in mobility and balance can be critical, as these make it difficult for older adults to perform daily tasks and negotiate the physical environment safely.

Older adults may be more sensitive to the cold, and home temperatures should be set at a minimum of 65ºF (18ºC) (Kercher & Rubenstein, 2002). Tap water burns occur disproportionately in older person, and when bathing can be serious or fatal if syncope or a fall occurs (Walker, 1990). Hot water heaters should be set no higher than 120ºF (48.9ºC) to avoid scalds and burns (Ehrlich, 2006; Kercher & Rubenstein, 2002). Specialized bath mats are available that change color when the water is too hot, providing an alternative visual cue. Temperature limiting mixer valves are available that shut the flow off when the water gets too hot.

Lapses in memory can also increase burn hazards from cooking, when cooking implements overheat, a forgotten pot boils over, or when a user fails to turn the stove off after use. Incorporating stay-cool handles on pots, pans, and implements, as well as additional sensory cues on appliances (such as an illuminated red warning symbol that stays lit as long as when a burner remains hot) are design features that minimize the potential for accidental burns.

Product designs also must account for the possibility of lower coordination, reduced grip strength, joint stiffness, and hand tremor (Pirkl, 1994). Hence, sliding controls and levers are easier to use than knobs that must be gripped and turned; larger diameter caps are easier to grip than small; and a well-designed flip top maybe easier to use than a screw top. To accommodate reduced touch perception and motor coordination, larger surfaces (e.g. for telephone dialing) are easier to actuate or depress than small ones; textured rather than smooth surfaces will increase the perception of features actuated by pressure; and redundant sensory cues help signal when something has been actuated or correctly placed into position (e.g. a “click” or tone when a key is depressed; a “snap” when something locks into position).

Tasks should require simple and discrete movements rather than complex or rapid sequences of movement (Vercruyssen, 1997). When a repetitive sequence of actions or a feedback response is employed, the user should be able to adjust them to a slower, more comfortable pace (e.g. adjustable response speeds on keyboards or adjustable tracking on a computer mouse or scroll bar) (Fisk, Rogers, Charness, Czaja, & Sharit, 2009c).

Attention must be paid to features of the physical environment that impede people with lower flexibility and coordination or those who have impaired sensory or cognitive functioning (Bakker, 2003; Byron, 2009; Newton, 2003). For example, unsecured objects (such of end-of-aisle displays, stacked packaging, swinging doors, etc.) present obstacles to older people. As noted earlier, shiny floors, particularly with sunlight on them, are a prime location for falls. Area rugs are a hazard unless they are non-skid or secured.

Color contrast helps to distinguish distinct surfaces (e.g. a contrasting strip on the edge of a step facilitates distinguishing one step from another.) Strong color contrasts can be used to help distinguish floors from walls, doorframes from adjoining walls, or furniture from flooring. Avoid strongly patterned carpeting, which creates visual–spacial difficulties (i.e., the appearance of relief or undulating surfaces) that can throw an older person off-balance. (For detailed guidance on environmental risk factors and home safety, as well as a bibliography of elder-safety checklists available on the Internet, the reader is referred to (Kercher & Rubenstein, 2002).)

Product placement within the environment should account for lower trunk heights in older people and their limitations in stretch and reach. For example, the height and distance of wall mounted items in the home or shelf placement of products in the retail environment should be adjusted so that it does not challenge and frustrate the older person (For an extensive discussion of anthropometry and human biometrics measurements across age groups, the reader is referred to Kroemer, 1997).

Accommodations discussed in this section are summarized in Tables S6 and S7 of the supplement.

### 3.8 Changes in Memory and Cognition

Researchers have identified various forms of memory:

*Working memory* is the active memory of what is currently perceived or thought about (reviewed in (Baddeley, 1992)). The capacity of working memory in young adults is about 7 chunks of information (Miller, 1956); and the length of time that stimuli are actively is about 30 seconds. The length of time that information is processed in active memory decreases with age (Salthouse, 1994). Although the impact on simple procedures is negligible, recall of multiple instructions or complex sequences of information becomes difficult. Fewer discrete pieces of information are held in active memory at any given time, making older adults susceptible to information overload and more easily overwhelmed by multi-tasking.

*Prospective memory* is the ability to remember to do something in the future. An older person finds it more difficult to remember to do something after a certain elapsed time (e.g. take pill every four hours) than to remember to do something tied to a specific prompt or event (e.g. once the buzzer sounds, turn off the oven) (Jager & Kliegel, 2008; Park, Hertzog, Kidder, Morrell, & Mayhorn, 1997).

*Semantic memory* is a person’s long-term repository of world knowledge (e.g. language, history, the arts, cultural norms) (Tulving, 1972). Barring a pathological condition, such as Alzheimer’s disease, long-term memory is well preserved as people age; indeed, the recognized expert is often an older, experienced person. Temporary blocks in retrieval can occur; an example is the “tip of the tongue” experience—the inability to instantly recall a particular term but retrieving it subsequently. The underlying repository of information, however, remains intact.

*Procedural memory* is the memory of how to carry out tasks learned in the past (Squire, 1986). The knowledge of how to perform automatic behaviors (such driving) or a well-practiced routine (such as following a recipe and cooking a meal) remains largely intact, even though such behaviors may be slower and more deliberate if they require attention and coordination. However, a previously automatic procedure is harder to inhibit if it is altered in a new context (e.g. expecting a clockwise turn to increase temperature or volume or presuming that placing a light switch in the up position will turn the light on).

Older people can transfer old skills to a new context (e.g. paradigms of computer software use from one program to another). They can learn new skills, but require more time, slower pacing, and more opportunities for practice and repetition to become proficient.

*Attention*, the capacity to maintain focus on a particular stimulus, changes with age. The older person is less able to inhibit competing information and is slower to orient their attention from one thing to another. Current research suggests that that the level of brain activity related to attending to a goal does not change with age; what diminishes is the ability to ignore competing sensory information unrelated to the goal. As a result, frequent though brief shifts in attention slow the older person’s speed of processing relevant information.

*Spacial cognition*, the ability to mentally orient in three dimensions based on other cues, is also more difficult for the older person.

Finally, language tends to be interpreted more literally. The older person may be less adept at drawing inferences when the cultural context is unfamiliar or when information is presented in a glib, sarcastic, or subtly ironic manner. Communication that employs such forms of humor, for instance, some forms of advertising, can be confusing and alienating to an older person.

The interested reader is referred to extensive reviews that discuss changes in attention, executive function, memory, language, and visual-spacial functioning (Drag & Bieliauskas, 2010; Fisk *et al.*, 2009a; Gazzaley, 2009; Howard Jr. & Howard, 1997). Changes in age-related performance on standardized cognitive tests are reviewed in (Salthouse, 2010).

A summary of memory and cognition changes discussed in this section is presented in Supplemental Table S8.

### 3.9 Accommodating Cognitive Changes

The keys to presenting information to older adults are (1) simplicity, (2) intuitive logic (e.g. consistency with established procedures), (3) moderate pace, and (4) a minimum of non-relevant information.

Visual information should be spare and uncluttered. One must highlight relevant information and minimize irrelevant and potentially distracting information. This is critical, for example, in prescription medication labeling and in computer interface design. Pictorial icons in particular should be tested with older audiences to ensure that they convey what is intended. Readability statistics should be performed on written text to be sure it is not overwhelming.

The presentation format (whether textual, pictorial, or auditory) should be simple and intuitive to minimize the possibility of misinterpretation. For example, medication usage instructions (dosage, side-effects) and warning labels should be formatted consistently and specific types of information placed in a predictable location. Similarly, in the case of information technology, such as computer programs, training materials, or web sites, the placement and style of computer commands and navigation buttons should be consistent. Frequent and important actions should be readily visible and accessible; error correction should be easy (“Undo” button) (For in-depth information specific to information technology and the older user, the reader is referred to Fisk *et al.*, 2009d, 2009f, 2009h).

Procedures should consist of simple, discrete steps consistent with established practices. Performing a sequence of several steps to achieve a single outcome, or requiring the processing of multiple pieces of data to draw a conclusion, is unnecessarily complex to the older person. Web site navigation can be particularly challenging without prompts that show where one is or what has been done at the particular point in the process (e.g. booking flights, shopping check-out procedures, etc.).

When presenting auditory information, simple instructions and brief messages are preferred. A moderate pace should be employed; key information should be repeated; contextual cues should be incorporated to emphasize connections and prompt recall. Redundant sensory cues will reinforce a correct action. Opportunities for practice and review are important.

The current trend toward cramming a vast array of features, displays, and controls into appliances, telephones, and automobile dashboards is counterproductive. Multiple displays are distracting and confusing to older users. If several features are included, the user should be able to choose whether or not to activate them, preferably with assistance at the point of sale.

Reviews of effective ways to convey information to the older user can be found in (Fisk *et al.*, 2009a; Howard Jr. & Howard, 1997; Siple, 2009). A summary of guidelines to accommodate changes in memory and cognition is presented in Supplemental Table S9.

## 4. The Importance of Respectful Inclusion

Aging should be viewed not as a liability or a form of pathology but as the common destiny of the fortunate. People in their 50s, 60s and 70s are active, engaged in their communities, and determined to remain relevant to the societies in which they live. Many older people feel years younger than their chronological age. Although they may notice aspects of diminished capacity, they do not view themselves as old. They want to remain independent, productive, and socially integrated.

Older people and people with a range of abilities appreciate products, communication materials, and features of the physical environment that are contemporary in design, accessible, and easy to use. Thoughtful design accommodates their needs in a way that does not segregate or stigmatize. It allows the user to understand instructions, to use a product or device as intended, to interact with the environment with minimal difficulty, and to avoid potential hazards. The accommodation guidelines presented here are consistent with the principles of Universal Design that promote equity and accessibility for people with diverse abilities (Table 1) (Gassman & Reepmeyer, 2008).

Researchers are encouraged to integrate the input of older adults into the design process (for guidance on recruiting representative populations, engaging the participants, gathering valid data, and statistical considerations, the reader is referred to (Fisk, Rogers, Charness, Czaja, & Sharit, 2009b, 2009g, 2009i; Schmidt-Ruhland & Knigge, 2008). Progress has been made in developing and validating new instruments to assess product and environmental usability based on universal design criteria (Beecher & Paquet, 2005; Fange & Iwarsson, 1999, 2007; Iwarsson, Horstmann, Carlsson, Oswald, & Wahl, 2009; Lenker, Nasarwanji, Paquet, & Feathers, 2011). These may serve as iterative tools to evaluate alternative designs and to assess the effectiveness of accommodation.

In summary, the thoughtful application of universal design principles leads to design that is inclusive and transgenerational: design that does not highlight functional limitations but instead is practical and appealing to people of all ages and differing abilities (Pirkl, 1994). Such design promotes public health and well being by minimizing hazards and by accommodating people with a range of sensory, physical, and cognitive function as active, integrated, and relevant members of society. 24
